# Supplementary material for: Comparative Analysis of Ultrasonography and MicroCT Imaging for Organ Size Evaluation in Mice
Source: J Imaging. 2025 Jun 18;11(6):200. doi: 10.3390/jimaging11060200 (PMC12194591; doi:10.3390/jimaging11060200)
Supplement: Supplementary file 1 [file jimaging-11-00200-s001.zip › jimaging-3562963-supplementary.pdf]

SUPPLEMENTARY DATA

Table S1. BIOLOGICAL DATA OF THE SAMPLE

Strain	Sex	Number of animals	Mean Weigh	Mean Age
**C57BL/6**	Female	9	21.88	126.33
**C57BL/6**	Male	1	27.7	391
**NOD.Cg-Rag1tm1Mom Il2rgtm1Wjl/SzJ**	Male	2	32.25	167
**NOD.Cg-Prkdcscid Il2rgtm1Wjl/SzJ**	Female	4	25.71	152.5
**Athymic Nude-Fox N1**	Female	4	31.13	227
**Athymic Nude-Fox N1**	Male	3	22.97	176.33

Table S1: biological data from the sample. Weigh is noted in grams and age in weeks.

Table S2. REFERENCE TISSUE DENSITIES

	Tissue Density
**Ovary (ICRU-44)**	1.050
**Spleen**	1.050
**Brain, Grey/White Matter (ICRU-44)**	1.040
**Breast Tissue (ICRU-44)**	1.020
**Heart**	1.053
**Kidney**	1.066
**Lung Tissue (ICRU-44)**	1.050
**Adipose Tissue (ICRU-44)**	0.950
**Blood, Whole (ICRU-44)**	1.060
**Testicles (ICRU-44)**	1.040
**Tissue, Soft (ICRU-44)**	1.060
**Tissue, Soft (ICRU Four-Component)**	1.000
**Muscle, Skeletal (ICRU-44)**	1.050

Table S2: Specific density of each organ. Density values in mg/mm^3

Table S3. STATISTICAL RESULTS OF ORGAN VOLUMES

Organ / imaging technique	Mean	Median	Range	Std Dev
**Heart CT Manual**	289.238	270.095	214.562	54.611
**Heart CT diameter**	160.967	162.200	114.791	34.548
**Heart sample**	139.918	130.342	130.389	36.197
**Heart US**	216.328	221.383	220.402	56.828
**Spleen CT Manual**	84.382	79.621	147.156	34.400
**Spleen CT diameter**	43.866	39.874	99.547	20.804
**Spleen sample**	97.683	82.667	237.524	56.017
**Spleen US**	89.366	85.444	233.380	53.787
**Left kidney CT Manual**	227.402	193.868	283.453	83.219
**Left Kidney CT diameter**	202.897	184.747	247.667	76.162
**Left Kidney sample**	212.675	201.126	240.150	59.674
**Left Kidney US**	179.179	169.202	152.810	42.930
**Right kidney CT Manual**	248.398	219.926	338.338	103.030
**Right Kidney CT diameter**	202.351	158.063	328.925	97.489
**Right Kidney sample**	217.487	213.415	252.345	63.241
**Right Kidney US**	180.937	178.246	192.154	49.502
**Left LN CT Manual**	3.503	2.558	18.107	4.554
**Left LN CT diameter**	2.736	1.299	19.994	4.221
**Left LN sample**	7.103	6.792	16.981	4.542
**Left LN US**	3.061	1.936	17.309	4.111
**Right LN CT Manual**	3.768	2.377	21.104	4.860
**Right LN CT diameter**	3.131	1.904	19.035	4.193
**Right LN sample**	6.604	5.377	17.453	4.52
**Right LN US**	3.858	2.163	31.872	6.603
**left Ovary CT Manual**	5.568	5.065	13.256	3.572
**Left Ovary CT diameter**	4.707	4.778	11.966	2.804
**Left Ovary sample**	9.73	9.524	10.476	3.562
**Left Ovary US**	4.462	4.204	8.906	2.622
**Right Ovary CT Manual**	5.013	4.003	10.466	3.080
**Right Ovary CT diameter**	4.789	4.530	6.142	1.708

**Right Ovary sample**	8.841	8.143	10.667	3.375
**Right Ovary US**	4.950	4.333	10.482	2.729
**Left Test CT Manual**	76.746	81.088	47.712	19.202
**Left Test CT diameter**	76.893	71.286	74.651	28.028
**Left Test sample**	80.593	80.288	62.308	21.865
**Left Test US**	73.387	73.859	49.307	25.292
**Right Test CT Manual**	70.554	78.227	50.825	20.989
**Right Test CT diameter**	72.286	70.047	75.707	29.744
**Right Test sample**	84.391	85.337	45.192	16.401
**Right Test US**	78.500	74.638	56.208	21.617

Table S3: Statistical results of the different organs and methods of imaging and quantification protocols. Mean, Median, Range and Standard deviation units are cubic millimeters.

Table S4. REQUIRED TIME FOR EACH ORGAN AND IMAGING TECHNIQUE

Organ	US imaging (min)	US diameter analysis (min)	microCT imaging (min)	microCT diameter analysis (min)	microCT manual analysis (min)
Heart	2	2	4	4	8
Spleen	2	2	4	6	5
Left Kidney	1	2	0	3	7
Right Kidney	1	2	0	3	7
Left ovary	1	1	0	2	3
Right ovary	2	1	0	2	3
Left lymph node	1	1	4	2	2
Right lymph node	1	1	0	2	2
Left testicle	1	2	0	4	10
Right testicle	1	2	0	4	10

Table S4: Required mean time for each organ scan and analysis mode. Values are in minutes and calculated as a means of the total samples analyzed.

Table S5. CORRELATION ANALYSIS OF ORGAN VOLUMES AND DIFFERENT IMAGING TECHNIQUES

HEART			
	**Ultrasound volume**	**uCT diameters method volume**	**uCT manual segmentation method volume**
**Ex vivo Sample Volume**	0.287	0.428	0.481
P value	0.247	0.0766	0.0433

SPLEEN			
	**Ultrasound volume**	**uCT diameters method volume**	**uCT manual segmentation method volume**
**Ex vivo Sample Volume**	0.824	0.774	0.845
P value	0.0000262	0.000264	0.0000104

KIDNEY							
	**Left Kidney Ultrasound volume**	**Left Kidney uCT diameters method volume**	**Left Kidney uCT manual segmentation method volume**	**Right Kidney Ex vivo Sample Volume**	**Right Kidney Ultrasound volume**	**Right Kidney uCT diameter**	**Right Kidney uCt manual segmentation method volume**
**Left Kidney Ex vivo Sample Volume**	0.564	0.789	0.733	0.987	0.813	0.713	0.836
P value	0.0183	0.0001	0.00123	3.38E-14	0.000072	0.00090	0.0000289
**Right Kidney Sample Volume**					0.822	0.67	0.807
P value					0.000051	0.00233	0.0000892

LYMPH NODE							
	**Left LN Ultrasound volume**	**Left LN uCT diameters method volume**	**Left LN uCT manual segmentation method volume**	**Right LN Ex vivo Sample Volume**	**Right LN Ultrasound volume**	**Right LN uCT diameter**	**Right LN uCT manual segmentation method volume**
**Left LN Sample Volume**	0.87	0.846	0.862	0.71	0.831	0.852	0.843
P value	0.000005	0.0000186	0.00000881	0.0014	0.000035	0.0001	0.0000212
**Right LN Ex vivo Sample Volume**					0.811	0.682	0.667

P value					<u>0.000077</u>	<u>0.0018</u>	<u>0.0025</u>

OVARY							
	**Left Ovary Ultrasound volume**	**Left Ovary uCT diameters method volume**	**Left Ovary uCT manual segmentation method volume**	**Right Ovary Ex vivo Sample Volume**	**Right Ovary Ultrasound volume**	**Right Ovary uCT diameter**	**Right Ovary uCT manual segmentation method volume**
**Left Ovary Ex vivo Sample Volume**	0.206	-0.0876	0.22	0.856	0.217	0.161	0.297
P value	0.521	0.787	0.491	<u>0.000385</u>	0.498	0.617	0.348
**Right Ovary Ex vivo Sample Volume**					0.429	0.305	0.402
P value					0.164	0.335	0.195

TESTICLE							
	**Left Testicle Ultrasound volume**	**Left Testicle uCT diameters method volume**	**Left Testicle uCT manual segmentation method volume**	**Right Testicle Ex vivo Sample Volume**	**Right Testicle Ultrasound volume**	**Right Testicle uCT diameter**	**Right Testicle uCT manual segmentation method volume**
**Left Testicle Ex vivo Sample Volume**	0.842	0.951	0.958	0.942	0.955	0.9	0.871
P value	<u>0.0353</u>	<u>0.00361</u>	<u>0.00265</u>	<u>0.00495</u>	<u>0.00304</u>	<u>0.0145</u>	<u>0.024</u>
**Right Testicle Ex vivo Sample Volume**					0.891	0.857	0.775
P value					<u>0.0173</u>	<u>0.0292</u>	<u>0.0705</u>

Table S5: Statistical correlations of the different imaging techniques and analysis methods in each organ. For each organ, the volumes obtained (Ex vivo sample, ultrasound and microCT measurements in both protocols: diameter measurement and manual segmentation) are compared. Ex vivo volumes are used as a gold standard to evaluate the accuracy of different imaging techniques and protocols for organ volume calculation. When possible, contralateral organs are compared to assess possible measurement errors of the different methods. Significant discrepancies between contralateral organ volumes in any of the imaging techniques and protocols could indicate an incorrect quantification protocol in the analyzed organ.

For each comparison, the value in the top row is the correlation coefficient and the bottom row corresponds to the P-value or significance of the comparison between results. A positive coefficient indicates that both

parameters are increasing or decreasing simultaneously. A negative coefficient indicates opposite trends, one parameter increasing while the other decreases. For P-values, values less than 0.05 are considered statistically significant, indicating a correlation between organ volumes. For these correlations, the lower the P-value, the more connected the volumes are. For P-values greater than 0.05, the two values compared are not statistically correlated. Statistically significant correlations have been underlined for better visualization of the results.
